# 152 Changing Urine Reflex Criteria and Rejecting Urine Samples for Culture with ≥10 Epithelial Squamous Cells in Urinalyses

**DOI:** 10.1017/ash.2026.10555

**Published:** 2026-06-23

**Authors:** Mohamad Shieb, Yu Kawai, Jose Simon Duran, Gina Rohlik, Brenda Schiltz, Anna Mujic, Kristin Cole, Vickie L. Miller, Leah Siple, Travis Kirkpatrick, Emily Levy

**Affiliations:** 1 Mayo Clinic; 2 Mayo Clinic Children’s; 3 Mayo Clinic Children’s

## Abstract

**Background:** Central line-associated bloodstream infections (CLABSIs) remain a challenge in pediatric intensive care units (PICUs); central line days have been directly correlated with risk of CLABSI. The Mayo Clinic PICU is a 23-bed multidisciplinary unit including dedicated bone marrow transplant beds with an average of 1048 admissions annually. In 2024, the unit’s CLABSI rate was 0.82 per 1,000 central line days or approximately 1–3 CLABSIs per year. Weekly multidisciplinary CLABSI Prevention Rounds were piloted from August 2024 to March 2025 with the aim of reducing central line device utilization rate as a method to reduce CLABSI risk. **Methods:** A multidisciplinary team of Clinical Nurse Specialists (CNS), Nurse Managers, and off-service PICU physicians conducted weekly CLABSI rounds on PICU beds (excluding 4 BMT beds), meeting with each bedside nurse as well as the on-service providers. The CLABSI team assessed central line necessity using a standard questionnaire and provided education to physicians and nurses regarding central line care and CLABSI prevention. Monthly data were compared for the same 8 calendar months pre versus post intervention for central line days, patient days, and device utilization rate (DUR; central line days/patient days), both unadjusted and adjusted for PRISM (Pediatric Risk of Mortality) 3 severity of illness score. Pre- and post-implementation staff surveys assessed confidence in line management and interdisciplinary communication using Likert scales. **Results:** Thirty-two CLABSI rounds with a total of 118 individual patients and 45 discrete interventions were noted over 8 months and compared to the 8 pre-intervention corresponding months. Pre versus post monthly DUR was 0.42 [0.32–0.57] vs 0.46 [0.30–0.55], p = 0.49 (PRISM 3–adjusted p = 0.97). Nursing confidence in ‘discussing central line need’ significantly improved post-implementation, 5 [4–5] vs 5 [5–5], p = 0.035. Nurses also positively perceived third-party involvement in need assessment, 4 [3–4] vs 4 [3–4], p = 0.06. All surveyed clinicians trended toward being less likely to think a central line was left in too long, 3 [3–4] vs 3 [2–4], p = 0.06. **Conclusion:** Structured weekly multidisciplinary CLABSI rounds are a feasible intervention that empowers bedside nurses and improves confidence in line maintenance and interdisciplinary communication. Median DUR was not significantly altered by the intervention, but nursing perception of central line management improved qualitatively in pre- versus post-implementation surveys. Continued expansion of multidisciplinary initiatives addressing central line necessity and management is needed to further evaluate overall effects on DUR and CLABSI incidence.

